# Impacts of ESOGER home-based care and health services on spousal caregivers' anxiety, quality of life and burden: Findings from a pilot randomized controlled trial

**DOI:** 10.1016/j.tjfa.2025.100114

**Published:** 2025-12-11

**Authors:** Olivier Beauchet, Camille Normandin, Pascal Mathieu, Kevin Galéry

**Affiliations:** a Departments of Medicine and Geriatrics, University of Montreal, Montreal, Quebec, Canada; b Research Centre of the Geriatric University Institute of Montreal, Montreal, Quebec, Canada; c Department of Medicine, Division of Geriatric Medicine, Sir Mortimer B. Davis Jewish General Hospital and Lady Davis Institute for Medical Research, McGill University, Montreal, Quebec, Canada; d Department of community health, Canadian Red Cross, Quebec, Canada

**Keywords:** Randomized controlled trial, Spousal caregiver, Home care and support services, Burden, Quality of life

## Abstract

**Background:**

Spousal caregivers of ill older adults face increasing risks of deteriorating mental health and burden. “Socio-Geriatric Evaluation” (ESOGER) is home-based care and health services for ill older adults. This study aimed to examine changes in anxiety, quality of life and burden over a 3-month period in spousal caregivers of ill older adults who benefits from ESOGER home health care and support services.

**Methods/design:**

A randomized controlled trial (RCT) with two parallel arms enrolled 42 spousal caregivers distributed equally between the intervention group and the control group. The intervention consisted of ESOGER, a telehealth-based home care program that evaluates older adults’ health and social needs and provides personalized recommendations and referrals to health and community services to ill spouses, implemented through the Canadian Red Cross. Spousal caregivers were assessed at baseline (M0) and at three months (M3). Anxiety was evaluated using a visual analogue scale (VAS) ranging from 0 (no anxiety) to 10 (severe anxiety) and the EuroQol-5D assessed quality of life using. Burden was measured using the 4-item Zarit scale.

**Results:**

Anxiety (*P* < 0.001) and burden (*P* = 0.003) increased significantly, and the quality of life decreased (*P* = 0.018) in the control group at M3 compared to M0. In the intervention group anxiety decreased significantly (*P* < 0.001) over the 3-months follow-up. Only burden was significantly lower in the intervention group compared to the control group (*P* = 0.022) at M3. The changes in scores of the 4-item Zarit scale between M0 and M3 (*P* = 0.011) and of the EQ-5D visual analogue scale (*P* = 0.024) were significantly different between groups, showing an improvement in the intervention group.

**Conclusion:**

This study highlights the positive impact of ESOGER home-based care on spousal caregivers, showing reduced anxiety and burden while improving quality of life. These findings underscore the importance of structured home care services in supporting caregivers' well-being and sustaining home-based care for older adults.

## 1 Background

Family caregivers are unpaid individuals who provide care and support services to a family member experiencing a loss of autonomy and/or independence [1]. This assistance is typically provided at home and encompasses a broad range of responsibilities, from personal care to managing legal and administrative matters [1,2]. The caregiver role may be both demanding and time-consuming, often taking a toll on the caregiver’s own mental health and quality of life [2,3]. When caregiving demands become overwhelming, it can lead to burnout [[2], [3], [4]]. Although caregiving can be associated with stress, anxiety, and burden, the literature also emphasizes its positive aspects [[1], [2], [3], [4], [5], [6]]. Many caregivers report a sense of satisfaction, fulfillment, and strengthened purpose in life through their role [6]. For example, caregiving can enhance the emotional bond with the care recipient, provide a sense of accomplishment, and foster resilience. These positive experiences, often described as “caregiving uplifts,” coexist with the challenges of caregiving and contribute to a more nuanced understanding of caregivers’ well-being [5,6].

The health status of older adults is often marked by an accumulation of morbidities, leading to functional impairments and disabilities that increase their need for home care and support services to age in place [7]. The number of ill older adults living at home and requiring family caregivers is significant and continues to grow due to demographic shifts and rising life expectancy [8]. In 2022, 8.4 million Canadians were family caregivers, with many providing care to elderly family members [9]. However, as the population ages, so do caregivers. In Quebec, older spousal caregivers represent approximately 20 % of family caregivers [2]. This group is particularly vulnerable to declining mental health, quality of life and, thus, to burnout [[10], [11], [12], [13], [14]]. Given these risks, it is crucial to address the specific challenges faced by older spousal caregivers and implement support measures to safeguard their health and quality of life.

Home care is the preferred option for supporting older adults with incapacities, making spousal caregivers essential in providing care and support services [[1], [2], [3], [4]]. The COVID-19 pandemic further intensified their responsibilities, as disruptions in the healthcare system led to reduced access to home health care and support services [13]. Given this, interventions aimed at enhancing home care must also prioritize maintaining or improving the health and functional status of spousal caregivers. Supporting their well-being is crucial to sustaining the autonomous living and quality of life of both the older adult and their caregiving spouse for as long as possible.

It has been reported that slowing down the progression of worsening health by implementing home health care and support services in older community dwellers using a telehealth tool known as “Socio-Geriatric Evaluation” (ESOGER) was possible [[14], [15], [16]]. ESOGER is a telephone-based telehealth tool developed at the onset of the COVID-19 pandemic to facilitate healthcare and support services for older adults living at home [16]. It evaluates mental, physical, and social health, offering personalized recommendations for tailored health and social interventions. The Canadian Red Cross-Quebec (CRC-Qc) launched during the pandemic period a care and support service “Seniors Proximity Project” which offers phone call support and community referral assistance to people aged 65 and older. Since the end of the first year of the pandemic, the CRC-Qc has utilized ESOGER to support older community residents in Montreal (Quebec, Canada). ESOGER is employed to contact older adults by phone, assess their needs, and implement and monitor relevant home health care and support services. The effects of ESOGER use on spousal caregivers of assisted older adults who benefits from ESOGER home health care and support services has never been examined. We hypothesized that ESOGER home health care and support services implemented for assisted older adults could improve the mental health and decrease burden of their spousal caregivers. This randomized controlled trial (RCT) aimed to examine changes in anxiety, quality of life and burden over a 3-month period in spousal caregivers of ill older adults who benefits from ESOGER home health care and support services.

## 2 Material and methods

### 2.1 Design

The study was designed as a randomized controlled trial (RCT) with two parallel arms: an intervention group and a control group. The intervention consisted of ESOGER home health care and support services implemented for ill older adults living at home with a spousal caregiver, delivered through the CRC-Qc program. Caregivers and their ill spouses in both groups were assessed at baseline (M0) and again at three months (M3). In the intervention group, older adults received their assessment results and benefited from the CRC-Qc intervention. However, there was no direct intervention for their spousal caregivers who did not receive their own assessment results. In the control group, only an assessment was conducted, and neither the older adults nor their caregivers received any feedback on the results. In this group, participants received no intervention other than their usual standard care. Standard care for this population consists of primary care follow-up with their family physician, and access to community health and social services upon request, but without the structured assessment and referral process provided by ESOGER. No additional home-based care or support services were implemented for the control group during the study period. Participants were randomly assigned to the intervention or control group using block randomization with a 1:1 allocation ratio. Randomization lists were generated using the N’Query randomization software. The random assignment sequence was generated by an independent biostatistician who was not involved in the recruitment, assessment, or analysis, thus ensuring allocation concealment. Randomization was performed at the level of the care recipients (i.e., ill older adults), who were enrolled in the main ESOGER trial. Spousal caregivers were therefore indirectly assigned to either the intervention or control group depending on their partner’s allocation. Caregivers themselves were not randomized independently. The research team members who enrolled participants had no access to the randomization sequence. This RCT is registered on ClinicalTrials.gov (project number NCT05099042) and adheres to the CONSORT guidelines for randomized controlled trials [17].

### 2.2 Population

To participate in the study, individual had to meet the following inclusion criteria: to be aged 70 and over, to be a spousal caregiver of an ill adult aged 70 and over who benefits from ESOGER intervention, to live at home with their assisted ill spouse in the administrative health and social service region of the “*Centre intégré universitaire de santé et de services sociaux Centre-Sud-de-l’Île-de-Montréal”* (CCSMTL) of Montreal, to understand French or English and to agree to participate. The exclusion criteria were concomitant participation in another experimental study and the need for acute medical care. Recruitment of spousal caregivers occurred through the CRC-Qc program in CCSMTL. All eligible caregivers were contacted by trained research staff after their assisted spouse was enrolled in the ESOGER trial and were invited to participate. Informed consent was obtained prior to study inclusion. Between December 17, 2021, and June 3, 2022, a total of 150 assisted ill spouses were recruited into a RCT, respectively 69 in the control group and 81 in the intervention group. These participants benefited from ESOGER home care and support services and were followed for three months. 42 spousal caregivers (21 in the control group and 21 in the intervention group) of the assisted ill spouses participating in the RCT agreed to participate in the present study. They received no intervention. They were assessed at M0 and at M3.

### 2.3 Baseline and follow-up assessments

The spousal caregivers in the intervention and control groups were assessed at M0 and again at M3. At baseline, information about age, sex, activities of daily living (ADL) and instrumental activities of daily living (IADL) [18,19], number of medications daily taken, and use of walking aid were collected. Polypharmacy was defined as ≥ 5 different medications per day. A measurement of psychological stress using a visual analogue scale (VAS) for anxiety was also performed and graded from 0 (signifying no anxiety) to 10 (representing severe anxiety). Caregiver burden was assessed using the 4-item Zarit scale and the quality of life with the EuroQol-5D (EQ-5D) [20,21]. This last tool is composed of a questionnaire examining physical health issues, with scores ranging from 0 (i.e.*,* no issue) to 25 (i.e.*,* worst issue), and a visual analogue scale (VAS) assessing self-perceived health, ranging from 0 (i.e.*,* worst health imaginable) to 100 (i.e.*,* best health imaginable). At the 3-month follow-up, an assessment was repeated using the same standardized scales.

### 2.4 Intervention

The ESOGER home care and support services constituted the intervention provided to ill older spousal who need home assistance. Their spousal caregivers did not directly receive home care or support services. The intervention consisted of two key components: a structured evaluation of the participant’s health and social needs followed by personalized recommendations for home health care and support services based on the assessment findings [[14], [15], [16]]. The ESOGER assessment is a concise questionnaire that delves into five distinct subdomains through closed-ended inquiries. These subdomains cover: 1) COVID-19 symptoms; 2) Frailty; 3) Measurement of psychological stress using a VAS for anxiety; 4) Evaluation of social isolation defined by the absence of home care and support services, and of contact with others (family, neighbors, friends, healthcare or social professionals) over the phone or in person. For each subdomain, a risk level categorized in low versus high was determined [14]. Furthermore, an overall risk level (i.e., low defined as one or no high-risk domains versus high as ≥ 2 high-risk domains) is computed to identify older adults exposed to the highest overall risk, prioritizing recommended actions accordingly. Recommendations for home health care and support services were provided for each risk level. The tailored plan for the implementation of home health care and support services was discussed with the participants and spousal caregivers to obtain their agreement. These services were implemented by the CRC-Qc.

### 2.5 Outcomes

The outcomes measured included the mean values and standard deviations of the VAS anxiety score, the 4-item Zarit scale, and the EQ-5D at M0 and M3. Additionally, changes in these scores over the 3-month period were calculated using the following formula:((M3-M0) /((M3+M0)/2)) x 100. This formula quantifies the relative change in scale scores, allowing for a standardized comparison of differences over time.

### 2.6 Standard protocol approvals, registrations, and patient consents

Participants were recruited after obtaining written informed consent for research. The local ethics committee of the recruitment centre (i.e., *Comité d’éthique de la recherche vieillissement-neuroimagerie* for the CCSMTL) approved the project.

### 2.7 Statistics

All participants who were randomized and completed baseline assessments were included in the analyses, following an intention-to-treat principle. No imputation of missing data was required, as all participants completed follow-up assessments. The baseline characteristics of participants and changes in outcomes scores were summarized using means and standard deviations (SDs) for continuous variables and frequencies and percentages for categorical variables, as appropriate. Between-group comparisons of outcomes at M0 and M3 were performed using independent *t*-tests (for normally distributed continuous variables) or Mann–Whitney tests (for non-normally distributed variables). Within-group changes between M0 and M3 were assessed using paired *t*-tests or Wilcoxon signed-rank tests, as appropriate. Categorical variables were compared using Chi-square tests. Changes in outcomes (percentage variation between M0 and M3) were also compared between groups using Mann–Whitney tests. All statistical analyses were performed using SPSS (version 27.0; SPSS, Inc., Chicago, IL).

## 3 Results

As shown in the Table 1, there was no significant differences between the control and the intervention group at baseline. Intragroup comparisons showed that there was a significant increase of anxiety score (*P* < 0.001) and 4-item Zarit score (*P* = 0.003), and a decreased in EQ-5D visual analogic score (*P* = 0.018) in the control group at M0 compared to M3 (Table 2). In contrast, only anxiety score decreased significantly between M0 and M3 in the intervention group (*P* < 0.001). Intergroup comparisons showed that the 4-item Zarit score was lower (*P* = 0.022) in the intervention group compared to the control group at 3-month of follow-up. The changes in scores between M0 and M3 for the 4-item Zarit scale (*P* = 0.011) and the EQ-5D visual analog scale (*P* = 0.024) were significantly different between groups (Table 3). In the intervention group, both scores decreased, whereas in the control group, they increased.

**Table 1:** Baseline characteristics of participants in control and intervention group (n = 42).

	Participants		P-Value*
	Control (n = 21)	Intervention (n = 21)	
Age (years)			
Mean±SD	73.7 ± 4.6	75.6 ± 5.1	0.536
> 85, n ( %)	0	1 [4,8]	0.311
Female, n ( %)	12 (57.1)	17 (81.0)	0.095
ADL score† (/6)			
Mean±SD	5.5 ± 0.8	5.7 ± 0.6	0.568
Abnormal score (<5)	4 (19.0)	1 (4.8)	0.153
IADL score (/4)‡, mean±SD			
Mean±SD	3.9 ± 0.4	3.8 ± 0.4	0.437
Abnormal score (<4)	3 (14.3)	5 (23.8)	0.432
Polypharmacy¶, n ( %)	2 (9.5)	5 (23.8)	0.214
Walking aid, n ( %)	3 (14.3)	3 (14.3)	1.000

SD: Standard deviation.

ADL: Activities of daily living.

IADL: Instrumental activities of daily living.

⁎ : Comparison based on unpaired t-tests or chi-squared, as appropriate.

† : Ranging from 0 (worst score, full dependent) to 6 (best score, independent).

‡ : Ranging from 0 (worse score, non-autonomous) to 4 (best score, autonomous).

¶ : Number of medications taken daily ≥ 5.

**Table 2:** Comparison of mental and burden scale scores at baseline and 3 months in the control and intervention groups (n = 42).

	Control (n = 21)			Intervention (n = 21)			P-Value†	
	M0	M3	P-Value*	M0	M3	P-Value*	M0	M3
Anxiety score (/10), mean±SD	3.2 ± 2.2	4.9 ± 2.6	<0.001	3.7 ± 2.1	3.4 ± 2.5	<0.001	0.453	0.086
4-item Zarit score (/16), mean±SD	4.6 ± 3.3	7.3 ± 3.0	0.003	5.4 ± 4.8	4.4 ± 4.1	0.131	0.723	0.022
EQ5D mean±SD								
Questionnaire (/25)	7.7 ± 2.7	6.8 ± 4.4	0.699	9.1 ± 3.0	8.7 ± 3.2	0.538	0.161	0.175
Visual analogic scale (/100)	77.1 ± 15.6	63.1 ± 25.3	0.018	68.5 ± 25.9	73.6 ± 18.0	0.346	0.417	0.172

⁎ : Comparison based Wilcoxon test.

† : Comparison based Mann-Whitney test M0: At baseline M3: At 3-month follow-up period Significant P-values (i.e., <0.05) in bold.

**Table 3:** Comparison of changes in score of clinical scales between baseline and 3-month period follow-up in control and intervention group.

Change* in score between baseline and 3-month period follow-up expressed in percentage	Control (n = 21)	Intervention (n = 21	P-Value†
Anxiety score, mean±SD	50.8 ± 102.2	−16.6 ± 117.9	0.055
4-item Zarit score, mean±SD	54.5 ± 72.1	−12.1 ± 117.8	0.011
EQ5D mean±SD			
Questionnaire	−30.9 ± 86.8	−4.6 ± 36.3	0.910
Visual analogic scale	−25.4 ± 43.1	13.7 ± 52.2	0.024

⁎ : Calculated following the formula: ((M3-M0) /((M3+M0)/2)) x 100.

† : Comparison based Mann-Whitney test Significant P-values (i.e., <0.05) in bold.

## 4 Discussion

The findings suggest that spousal caregivers of ill partners who did not receive the home intervention experienced a significant increase in anxiety and burden, along with a decline in quality of life. In contrast, caregivers in the intervention group showed a notable reduction in anxiety. Additionally, burden and quality of life improved in the intervention group but deteriorated in the control group.

ESOGER home care and support services significantly alleviated anxiety among older spousal caregivers in our RCT. By assisting with daily caregiving tasks, home programs like ESOGER may reduce the emotional strain on caregivers, thereby decreasing their anxiety levels. For instance, the Resources for Enhancing Alzheimer's Caregiver Health (REACH) II intervention found that participants, recruited in this RCT and who received home care and support services, had better emotional health with their caregiving duties compared to those in the control group [22]. In addition, access to home care and support services allows caregivers to take breaks, promoting mental health [23]. These breaks not only provide time for rest but also enable caregivers to engage in social and recreational activities that enhance psychological resilience [[22], [23], [24]]. Furthermore, many home care programs offer counseling services, equipping caregivers with coping strategies and emotional support, which have been associated with reductions in anxiety [24,25]. Another key aspect is the improved health outcomes of the care recipient due to professional assistance. When caregivers observe their loved ones receiving quality care and experiencing fewer crises, their stress and anxiety levels naturally decrease [[22], [23], [24], [25]]. Collectively, these factors underscore the effectiveness of home care and support services in enhancing the mental health of older spousal caregivers.

The findings of our RCT also show a significant decrease in burden. The burden experienced by spousal caregivers can be significantly alleviated through professional assistance [24]. The explanations provided for the improvement in mental health may be applied to burden reduction, but additional specific mechanisms may be considered. First, professional caregivers take on many of the daily caregiving tasks, reducing the number of responsibilities that fall on the spousal caregiver. This reduction in workload directly decreases the physical and emotional burden of caregiving [[22], [23], [24]]. Second, with professional help, spousal caregivers can better manage their time, balancing caregiving duties with personal activities. This helps reduce role overload and the sense of being overwhelmed by caregiving responsibilities. Indeed, it has been demonstrated that time management improvements from professional care services led to significant reductions in caregiver burden [24,25]. Third, professional services often connect spousal caregivers with support networks and resources, providing practical advice and emotional support. This connection helps caregivers feel less isolated and more supported in their role [[23], [24], [25]]. These combined factors not only reduce burden but also create a more sustainable caregiving experience.

The results also showed a significant improvement in the quality of life of spousal caregivers. The decrease in anxiety and the reduction of caregiver burden directly contributes to better quality of life. As previously discussed, alleviating the physical, emotional and psychological demands of caregiving allows caregivers to focus more on their well-being [[21], [22], [23], [24], [25], [26], [27]]. Additionaly, professional caregivers are trained to deliver high-quality care, ensuring that the needs of the assisted person are met more effectively. This can lead to better health outcomes for the care recipient and indirectly benefit the caregiver by reducing stress and worry. For instance, Gaugler et al. (2009) indicated that high-quality professional care can improve the mental health of caregivers by reducing the frequency of health crises and emergencies [26]. Moreover, by assuming some caregiving responsibilities, professional caregivers provide spousal caregivers with more time to engage in self-care activities, hobbies, and social interactions, all of which are essential for maintaining mental health. According to a study by Zarit et al. (1998), home care and support service may allow caregivers to take breaks and thus showed to reduce caregiver stress and improve overall well-being [28]. It has been also shown that professional caregivers often provide spousal caregivers with information and resources about managing specific health conditions, which empowers caregivers and reduces feelings of helplessness. For instance, providing caregivers with knowledge and skills significantly improves their competence and confidence in caregiving roles [29]. Finally, knowing that a professional is involved in the care of their loved one can provide significant peace of mind to spousal caregivers, reducing anxiety and improving overall quality of life. According to Ducharme et al , the involvement of professional caregivers alleviates the constant worry experienced by many spousal caregivers, leading to better mental health outcomes [30]. These interconnected benefits highlight how home care and support services comprehensively enhance caregivers' overall well-being, ensuring sustainable and effective caregiving experiences. Although ESOGER was initially developed during the COVID-19 pandemic, its relevance extends beyond this context. The challenges faced by spousal caregivers, such as anxiety and decreased quality of life, are not specific to the pandemic. The structured evaluation and referral model of ESOGER remains applicable to routine home care and support services for older adults in non-pandemic settings.

The RCT design, the standardization of ESOGER home care and support services, and the absence of baseline differences between the intervention and control groups were key strengths of our study. However, several limitations should be considered. First, the RCT was conducted exclusively among older dyads living in Montreal, limiting generalizability. Second, as a pilot study with a small sample size, the study lacked statistical power. Third, precisely monitoring the level of "control" over the control group was challenging. Over the study period, participants in the control group may have received external assistance, potentially influencing the outcomes.

In conclusion, this pilot RCT demonstrates the significant benefits of the ESOGER home-based care and health services for spousal caregivers of ill older adults, highlighting the importance of structured home care interventions in supporting not only older adults but also their caregiving spouses. The structured evaluation, personalized recommendations and implementation of home health services provided spousal caregivers with crucial support, reinforcing the sustainability of home-based care. The results provide promising evidence supporting the integration of home care programs into caregiving strategies. Future research should explore the long-term impact of such interventions on caregivers' mental and physical health, as well as their effects on care recipients' outcomes. Additionally, scaling up and adapting these services to diverse caregiving populations could further validate their effectiveness. Ultimately, recognizing and addressing the needs of spousal caregivers is essential to ensuring both their well-being and the quality of care provided to ill older adults.

## Author contributions

Conceived and designed the experiments: Olivier Beauchet, Kévin Galéry

Performed the experiments: Kévin Galéry, Camille Normandin, Pascal Mathieu

Analyzed and interpreted the data: Olivier Beauchet

Contributed reagents, materials, analysis tools or data: Olivier Beauchet, Kévin Galéry, pascal Mathieu,

Writing of the manuscript: Olivier Beauchet

Revision of manuscript: Kévin Galéry, Camille Normandin, Pascal Mathieu

## Data statement

Data will be made available upon request sent by email to Dr Beauchet (olivier.beauchet@umontreal.ca).

## Declaration of generative AI and AI-assisted technologies in the writing process

No generative AI and AI-assisted technologies have been used to write the manuscript

## Funding

The study was funded by the Fonds de Recherche du Quebec (FRQ; 2021–0QBA-297388). The sponsor had no role in designing and conducting the study, nor in the collection, management, analysis, and interpretation of the data, nor in the preparation, review, or approval of the manuscript.

## CRediT authorship contribution statement

**Olivier Beauchet:** Writing – original draft, Project administration, Methodology, Investigation, Funding acquisition, Formal analysis, Conceptualization. **Camille Normandin:** Writing – review & editing, Project administration, Investigation. **Pascal Mathieu:** Writing – review & editing, Resources, Project administration, Investigation. **Kevin Galéry:** Writing – review & editing, Project administration, Methodology, Conceptualization.

## Conflict of interest

The authors declare no conflict of interest.
